# Clinical Efficacy of a Novel Titania Nanoparticle-Reinforced Bonding Agent in Reducing Post-Restorative Sensitivity: A randomized clinical trial

**DOI:** 10.12669/pjms.40.7.8779

**Published:** 2024-08

**Authors:** Nehal Amir, Afsheen Mansoor, Nouman Noor, Khadim Hussain

**Affiliations:** 1 Dr. Nehal Amir, BDS. Postgraduate Resident, Department of Operative Dentistry & Endodontics, School of Dentistry, Shaheed Zulfiqar Ali Bhutto Medical University, Islamabad, Pakistan; 2 Dr. Afsheen Mansoor, Ph.D. Associate Professor, Department of Dental Material Sciences, School of Dentistry, Shaheed Zulfiqar Ali Bhutto Medical University, Islamabad, Pakistan; 3 Dr. Nouman Noor, FCPS. Associate Professor, Head of Department, Department of Operative Dentistry & Endodontics, School of Dentistry, Shaheed Zulfiqar Ali Bhutto Medical University, Islamabad, Pakistan; 4 Khadim Hussain, Ph.D. Assistant Director CRS Agriculture Department, Islamia University, Bahawalpur, Pakistan

**Keywords:** Composite resins, Dentin bonding agents, Dentin Sensitivity, Nanoparticles, Titania

## Abstract

**Objective:**

To evaluate the clinical efficacy of novel titania-nanoparticle reinforced bonding agent on post-restorative sensitivity in patients.

**Methods:**

This triple-blinded, randomized clinical trial included participants (n = 60) having Class- I and II cavitations with a minimum cavity depth of 3mm at Department of Operative Dentistry & Endodontics, School of Dentistry, Shaheed Zulfiqar Ali Bhutto Medical University, Islamabad from January 5, 2023, to June 20, 2023. They were randomly assigned into two groups A and B (n = 30). After informed consent, restorative intervention was accomplished using an etch-and-rinse adhesive strategy. In Group-A, titania-nanoparticle-incorporated bonding agent was used for composite restoration, while in Group-B, bonding agent without nanoparticles was used. The primary outcome was assessed using Visual Analogue Scale mean score. Participants were instructed to rate their sensitivity status at follow-ups: 24 hours, one week, and one month. Mann-Whitney U test was employed to compare sensitivity between the two groups.

**Results:**

According to results of this trial, a significant difference was observed between two groups after 24 hours (p = 0.004) and one week (p = 0.002). However, no discernible difference was observed after one month (p = 0.643).

**Conclusion:**

Post-restorative sensitivity in patients with composite restorations was reduced using titania-reinforced bonding agents as compared to bonding agents without nanoparticles. This shows that inclusion of titania nanoparticles into adhesive dentistry could be beneficial in resolving post-restorative sensitivity occurring with composite restorations.

## INTRODUCTION

The inception of nanotechnology has revolutionised clinical dentistry through its multitudinous contributions to biomaterial science. By embodying principles of nanoscience, it has not only ameliorated dental biomaterials but has also been considered a boon to modern clinical practise.^1^ Nanoparticles are cynosure of nanoscience, where atoms or molecules assemble as nanostructured agglomerates to impart superlative physiochemical and optical-magnetic attributes to materials.^2^ Within the prodigious lineage of metal-oxide nanoparticles, titanium dioxide (titania) has gained immense popularity owing to its biocompatibility, exceptional optical-mechanical properties, cost-effectiveness, and tremendous antibacterial potential.^3^

With the introduction of adhesive materials in restorative dentistry, paradigm has shifted to minimally invasive dentistry. Composite resin, being an eminent adhesive material, has primarily substituted dental amalgam as preferred material for posterior restorations.^4^ Adhesion to dental substrate, conservation of sound tooth structure, and excellent esthetics are some of the striking attributes of composite restorations. Nonetheless, polymerization shrinkage, poor wear resistance, and its hygroscopic nature jeopardize long-term survival of restoration.^5^

Polymerization shrinkage, besides compromising clinical longevity of restoration, produces many unpropitious consequences like cuspal deflection, post-restorative sensitivity, secondary caries, marginal staining, and microleakage at tooth-restorative interface.^6^ The potential sealing ability of bonding agents to shield patent dentinal tubules at dentin-adhesive interface can overcome this complication. However, this attribute of bonding agent is subjected to time-dependent decline, mainly due to the long-term instability and deterioration of hybrid layer.^7^,^8^

By fortifying bonding agents with nanoparticles, significant enhancements in physiochemical attributes and long-term stability of dentin-adhesive interface can be achieved.^9^ The rationale of this study was to upgrade the mechanical atributes of bonding agent by the addition of titania nanoparticles to reduce the issues of sensitivity associated with composite restorations. The hypothesis stated that occurrence and severity of post-restorative sensitivity in composite restorations were lessened using titania-incorporated bonding agents as compared to bonding agents without nanoparticles.

## METHODS

This trial is in conformance with the Consolidated Standards of Reporting Trials (CONSORT) 2010 guidelines. This was devised as a triple-blinded, parallel-group, randomized clinical trial with a 1:1 allocation ratio.

### Ethical Approval:

It was approved by Institutional Ethical Research Committee (SOD/ERB/2022/14)

The study was performed at Department of Operative Dentistry & Endodontics, School of Dentistry, Shaheed Zulfiqar Ali Bhutto Medical University, Islamabad, from January 5, 2023, to June 20, 2023. This interventional study is registered at ClinicalTrials.gov (ID: NCT05744648). All participants were duly briefed about the intervention along with its probable merits and demerits. A written consent form was ratified by each participant.

### Inclusion Criteria:

Participants must be 18 years old with good general health status. The nominated teeth should be vital, periodontally sound, and have established occlusion with natural or prosthetic antagonists and adjacent teeth. Carious lesions (Class-I and II having minimal cavity depth of 3mm, not exceeding 5mm) and prior faulty restorations should be designated for this intervention.

### Exclusion Criteria:

Participants with temporomandibular disorders, compromised oral hygiene, and those taking anti-inflammatory, analgesic, or psychotropic drugs. Teeth with periapical or periodontal pathology, previous endodontic treatment, and history of spontaneous pain were excluded.

By using WHO calculator, sample size was calculated to be 60 (30 participants in each group). Level of significance was 5%, and the power of the test was 80%. Test value for population mean was 1.38, while predicted population mean was 3.69, and population standard deviation was 1.6.^8^

Consecutive non-probability sampling method was utilised in this trial. Participants satisfying inclusion criteria were filtered from Outpatient department and were randomly distributed by Trial-autonomous researcher in each interventional group. An opaque, sealed envelope with designated coding for each group was used for allocation concealment. On account of triple-blinded nature of this clinical trial, participants, operator, and data analyst were kept blinded regarding designated groups. Bonding agents utilized in this intervention had identical bottles labelled with assigned codes (A and B) for each experimental group. A trial-autonomous researcher performed both blinding and allocation concealment in this intervention.

### Synthesis and Characterization of Titania nanoparticles:

Synthesis of nanoparticles was performed at Department of Dental Material Sciences, School of Dentistry, Shaheed Zulfiqar Ali Bhutto Medical University, from medicinal plant *Mentha spicata*.^10^ These nanoparticles were characterized for their dimensions, configuration, surface texture, phases, and fundamental constitution in *Materials Division, National Institute of Lasers and Optronics, Islamabad*. The characterization of these nanoparticles delineated a spherical shape, smooth surface, and a pure anatase phase with particle size of about 39nm. Elemental and functional constitutions were illustrated by pure titanium and oxygen peaks.^11^

### Inclusion of Nanoparticles into Bonding Agent:

Commercially available bonding agent Meta P & Bond (Meta Biomed, Korea) was employed for this trial, which was reinforced with titania nanoparticles at a mass fraction of 20% (1g of nanoparticles were added to a 5g bottle of bonding agent).^12^

### Clinical procedure:

Before clinical intervention, comprehensive medical and dental histories were recorded. The confirmation of carious lesion was done using a methodical approach. To ensure the patient’s comfort during procedure, tooth was locally anaesthetized using Lignospan Special-lidocaine hydrochloride 2% with 1:80,000 epinephrine (Septodont, USA). Under rubber dam isolation, carious lesions were excavated using high-speed handpiece. Depth of prepared cavity was estimated using periodontal probe. In case of class II cavitations, Palodent Plus Sectional Matrix System (Dentsply Sirona, USA) was applied before restorative intervention.

Subsequently, acid etching was performed using Meta Etchant 37% phosphoric acid (Meta Biomed, Korea) for 15 seconds, followed by rinsing and air-drying using air-water coolant. The successive steps were undertaken following principles of adhesive dentistry. Etched surface was coated with bonding agent Meta P & Bond (Meta Biomed, Korea) using micro brush for 20 seconds rigorously. This was followed by another application for 20 seconds. Adhesive coating was light-cured using LED curing light for 40 seconds. In *Group-A*, bonding agent incorporated with titania nanoparticles was used, while in *Group-B*, bonding agent without nanoparticles was used.

Afterwards, nanohybrid composite resin Nexcomp (Meta Biomed, Korea) was placed using incremental technique and light-cured for 40 seconds. After rubber dam removal, restoration was evaluated for premature contacts, and finishing was performed. Polishing was accomplished using Jiffy Original Composite System (Ultradent, USA).

### Outcome Assessment using Visual Analogue Scale (VAS) Mean Score:

**Fig.1:**
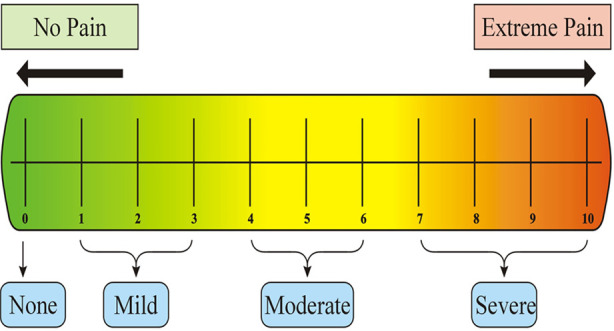
Visual Analogue Scale (VAS) Mean Score.

Using VAS mean score, sensitivity was evaluated after 24 hours, one week, and one month. Visual analogue scale is a measuring tool for unquantifiable personalised attributes with markings from 0 to 10 on a scale of 10 cm. It was calibrated for mean sensitivity scores of 0 (none), one to three (mild), four to six (moderate), and seven to ten (severe)^13^ (Fig.1). Participants were instructed to rate their sensitivity status following restorative intervention. Moreover, they were directed to specify whether pain was spontaneous or stimulated, and if stimulated, mention triggering factor.

### Statistical Analysis:

Data analysis was accomplished using SPSS 26 software. According to employed normality tests: Kolmogorov-Smirnov and Shapiro-Wilk tests, data for both groups at all time points departed significantly from normality. A nonparametric Mann-Whitney U test was applied to compare sensitivity between the two groups at follow-up periods. The level of significance was calibrated at ≤ 0.05.

## RESULTS

**Fig.2:**
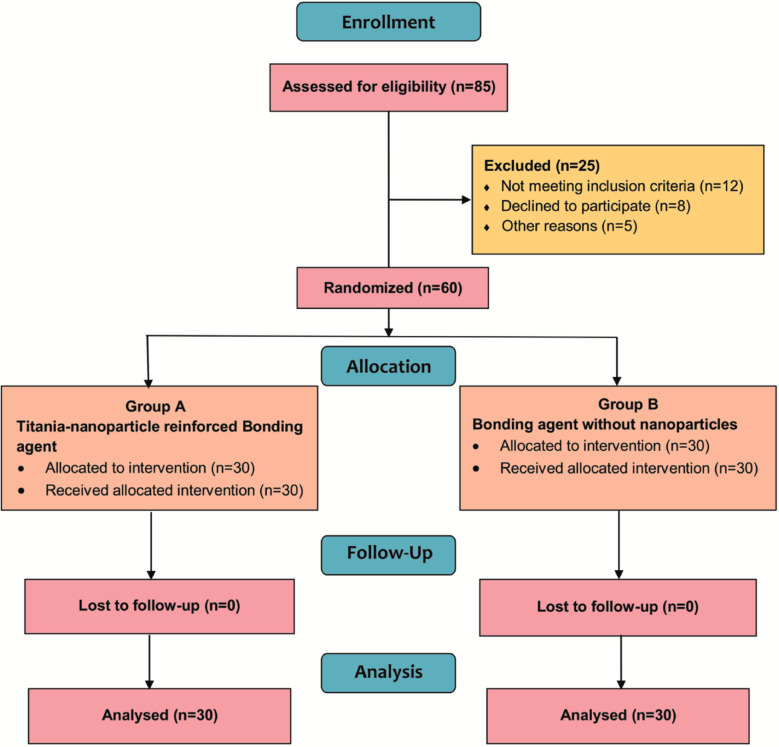
CONSORT Flow diagram.

This trial enrolled 85 participants. Out of these, 12 participants did not satisfy inclusion criteria, eight patients refused participation, and five participants had other reservations about this trial (Fig.2). Intervention comprised of 60 participants assigned to two groups (n= 30).

**Fig.3:**
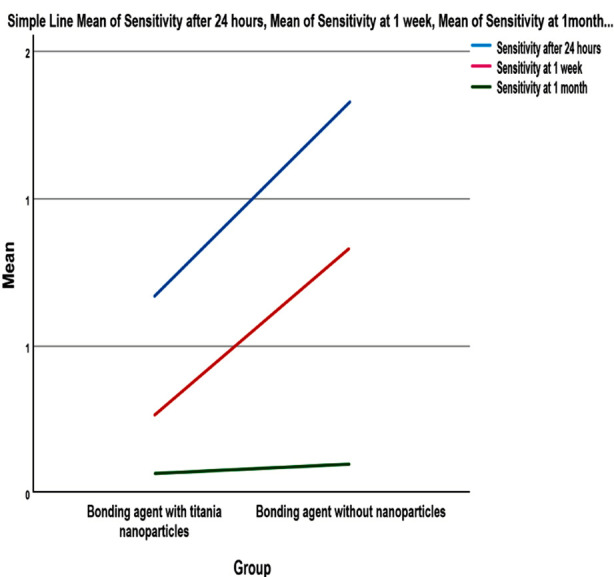
Variation of mean sensitivity patterns at various time points as mean line graph.

Characteristic attributes of involved participants and nominated teeth are shown in Table-I while Table-II delineates mean, standard deviation and 95% confidence interval values. The results of Mann-Whitney U test manifested significant difference in sensitivity between the two groups after 24 hours and at one week. However, after one month of restorative intervention, there was no discernable difference between two groups (Table-III). This has been illustrated as mean line graph (Fig.3).

**Table-I:** Characteristics of participants and nominated teeth.

Features		Group-A Titania-nanoparticle reinforced bonding agent	Group-B Bonding agent without nanoparticles (control group)
Gender	Male	16	16
	Female	14	14
Tooth distribution	Premolars	12	13
	Molars	18	17
G.V. Black’s classification	Class I	14	16
	Class II	16	14
Reason for restorative intervention	Primary or secondary caries lesion	17	21
	Marginal discoloration	5	3
	Marginal fractures	8	6

**Table-II:** Values of mean, standard deviation, and 95% confidence interval for the compared groups at three evaluation periods.

Group	Evaluation period	Mean	Standard deviation	95% Confidence interval	

				Lower limit	Upper limit
A	After 24 hours	0.67	0.758	0.38	0.95
	At 1 week	0.27	0.450	0.10	0.43
	At 1 month	0.07	0.254	-0.03	0.16
B	After 24 hours	1.33	0.884	1.00	1.66
	At 1 week	0.83	0.747	0.55	1.11
	At 1 month	0.10	0.305	-0.01	0.21

**Table-III:** Comparison of post-restorative sensitivity between titania-nanoparticle reinforced bonding agent and bonding agent without nanoparticles at three evaluation periods.

Evaluation periods	Groups	n	Mean ranks	Sum of ranks	Mann- Whitney U	p-value
After 24 hours	A	30	24.33	730.00	265.000	0.004
	B	30	36.67	1100.00		
At 1 week	A	30	24.20	726.00	261.000	0.002
	B	30	36.80	1104.00		
At 1 month	A	30	30.00	900.00	435.000	0.643
	B	30	31.00	930.00		

*p-value 0.05. Mann- Whitney U test.

## DISCUSSION

Resin composites bear an illustrious designation among contemporary restorative materials. By embodying principles of minimal intervention and adhesive dentistry, it has transfigured clinical practice. Nonetheless, it has several shortcomings, one of which is sensitivity. Hydrodynamic theory has elucidated sensitivity as an unpleasant sensation following fluctuations in tubular fluid flow upon stimulation, causing pressure variations leading to activation of terminal axonal fibres within pulp. This is mainly accredited to phenomenon of Nano-leakage, which occurs due to deficient penetration of adhesive into tubular network of demineralized dentin.^14^,^15^

Since its emergence in Mesopotamian era, nanotechnology has blossomed over past few decades. The pragmatic strategy concerning nanomaterials has encompassed disciplines of restorative dentistry, endodontics, pedodontics, oral surgery and dental implantology. Fundamentals of nanotechnology can be rationally employed to resolve issues of sensitivity with resin composites. Nanoparticles, when incorporated into bonding agents, not only magnify their physicochemical properties but also enhance longevity of hybrid layer by reducing nano-leakage at dentin-adhesive bonding interface.^16^ In this study, bonding agent was reinforced with novel biogenically synthesized titania nanoparticles. A study conducted by Mansoor A et al.^11^, demonstrated these nanoparticles procured from *Mentha spicata* to be biocompatible and ecologically secure. Additionally, various studies proved marked improvement in degree of conversion and mechanical attributes of bonding agents when titania was utilized as reinforcing fillers.^17^,^18^

In current study, nanoparticles were mixed into bonding agents at mass fraction of 20%.^12^ This was accomplished by considering the fact that fillers have profound impact on viscoelasticity and flowability of adhesives into dentinal tubules.^8^ Henceforth, percentage of incorporated nanoparticles was deliberately set at moderate levels to avoid any detrimental influence on adhesives while upgrading their properties.

In this trial, selected cases with class I and class II cavitations had minimum cavity depth of 3mm. This was consistent with findings of research that shows cavity depth does not influence post-restorative sensitivity.^19^ Additionally, etch-and-rinse adhesive strategy was employed in this study, keeping in mind that etch-and-rinse technique has greater occurrence of post-restorative sensitivity than self-etch strategy.^8^,^20^ Henceforth, influence of nanoparticle incorporation in bonding agents on sensitivity can be better judged.

Results of this study were analogous to the findings that incorporation of nanoparticles in bonding agent can significantly reduce sensitivity associated with composite restorations.^8^ Prevalence and severity of sensitivity subsequently to adhesive restorations were significantly less after 24 hours and one week as compared to control group while sensitivity records after one month for both groups were insignificant.

The hallmark of this trial lies in its novelty. No research work has been performed beforehand on titania-reinforced bonding agents and their impact on sensitivity of resin composites. Furthermore, this study incorporates eco-friendly and biogenically procured titania nanoparticles.^21^ This trial by reinforcing bonding agents with nanoparticles, can greatly minimise the drawbacks of posterior composites. Reinforcement of bonding agents with nanoparticles of *39nm* dimensions magnifies penetration of adhesive into tubular network of dentin, thus minimising post-restorative sensitivity.^11^ The triple-blinded study design further validates clinical results of this trial and renders it unbiased.

### Limitations:

This trial constituted an etch-and-rinse adhesive strategy owing to its greater implication on sensitivity.^20^ However, clinical findings of various studies show that adhesive technique (self-etch or etch-and-rinse) has no impact on sensitivity associated with composite restorations.^19^,^22^ The consequences of variables like cavity design, cavity depth, and placement techniques on post-restorative sensitivity were not considered in this trial. The one-month study duration was set in accordance with its feasibility for participants, although increasing the evaluation period could enhance validity and authenticity of this trial.

This study can contribute enormously to clinical dentistry by resolving long-established drawbacks of composite restorations. By reinforcing bonding agents with titania-based fillers, dental practitioners can provide high-quality restorative care to patients through provision of an aesthetic and durable restoration with superior physicochemical attributes. Nonetheless, this domain warrants further research work in future to support the evidence.

## CONCLUSION

Post-restorative sensitivity in patients with composite restorations was reduced using titania-reinforced bonding agents as compared to bonding agents without nanoparticles. This concludes that fortifying bonding agents with biogenically procured titania nanoparticles could be beneficial to adhesive dentistry. By resolving sensitivity in posterior composites, it can immensely serve patients by providing high-quality dental care to them.

### Authors` Contribution:

**NA:** Conception, methodology, data collection, preparing the manuscript.

**AM:** Methodology, supervision, critical review of manuscript, responsible for integrity of manuscript.

**NN:** Supervision, revision and approval of final version of manuscript.

**KH**: Data analysis and interpretation.
